# Extrusion-Based 3D Printing of Germinated Brown Rice Gel: Multi-Response Optimization of Process Parameters for Printing Fidelity and Hardness

**DOI:** 10.3390/foods15213979

**Published:** 2026-11-06

**Authors:** Liuyang Shen, Huiyu Zhang, Ran Liu, Yuhan Zhang

**Affiliations:** 1 College of Mechanical and Electronical Engineering, Tarim University, Alar 843300, China; feiyanghero@163.com; 2 Modern Agricultural Engineering Key Laboratory at Universities of Education Department of Xinjiang Uygur Autonomous Region, Tarim University, Alar 843300, China; 3 College of Agricultural Equipment and Energy Engineering, Northeast Agricultural University, Harbin 150030, China; s2507022090@neau.edu.cn

**Keywords:** 3D food printing, nozzle diameter, infill density, outer layer quantity, response surface methodology

## Abstract

Germinated brown rice (GBR) is a nutrient-dense whole grain, yet its coarse texture limits consumer acceptance. Three-dimensional (3D) food printing offers a promising strategy for structural reconstruction and value-added processing of GBR. This study investigated the effects of process parameters (nozzle movement speed, outer layer quantity, infill density, and nozzle diameter) on the printing fidelity and hardness of GBR gel. Single-factor experiments were conducted to determine reasonable parameter ranges, and response surface methodology was then used to model parameter interactions and optimize the process settings. In the single-factor tests, all four parameters significantly affected the two responses. Infill density and nozzle diameter were the dominant factors governing hardness, whereas printing fidelity was governed primarily by infill density, with nozzle diameter acting through significant quadratic and interaction effects. Numerical optimization identified the optimal settings as a nozzle movement speed of 25 mm/s, an outer layer quantity of 4, an infill density of 76%, and a nozzle diameter of 1.5 mm, achieving 94.64% printing fidelity and 314.16 g hardness with prediction errors below 2%. These findings establish a quantitative parameter framework for 3D food printing of this GBR gel and provide a reference for other cereal-based gel systems.

## 1. Introduction

Germinated brown rice (GBR) is recognized as a nutrient-dense whole grain, rich in γ-aminobutyric acid, polyphenols, and vitamins, and has garnered considerable attention from health-conscious consumers [1,2]. Nevertheless, the coarse texture and inferior palatability of GBR relative to polished rice severely constrain its acceptance as a staple food [3]. In this context, three-dimensional (3D) food printing has emerged as a processing strategy for the structural reconstruction and precision manufacturing of GBR, enabling the fabrication of customized geometries that may enhance sensory appeal and nutritional functionality [4]. However, in 3D food printing, the printing performance and quality of the final products are determined not only by intrinsic factors such as the gel properties (e.g., rheological properties) and formulation composition of the material to be printed, but also by extrinsic factors such as the printing process parameters [5,6].

Among the extrinsic variables, nozzle diameter, nozzle movement speed, infill density, and outer layer quantity constitute the principal determinants of deposition behavior and product quality in extrusion-based 3D food printing [6]. Nozzle diameter determines the thickness of the extruded filament and the interlayer bonding area; although a smaller nozzle refines the deposited lines and improves printing resolution, it increases extrusion resistance and the likelihood of discontinuous extrusion or even nozzle blockage, whereas an excessively large nozzle promotes material spreading and shape diffusion, thereby weakening boundary clarity and molding accuracy [7,8,9]. Nozzle movement speed affects the stability of the printing process: an excessively high speed causes insufficient material deposition, filament breakage, and poor interlayer stacking, whereas an excessively low speed leads to material accumulation, local bulging, and surface defects [10,11]. In addition to these nozzle-related parameters, the printing performance is also influenced by parameters related to the 3D model to be printed, such as infill density and outer layer quantity. Infill density describes the degree of filling inside the printed sample and directly reflects its internal support condition; it affects the internal structural compactness and mechanical strength of the sample and is closely related to the mechanical properties and shape retention ability of the printed product [12,13]. Likewise, outer layer quantity reflects the overall support and smoothness of the outer periphery of the printed model; the more outer layers there are, the smoother and stronger the outer wall becomes [14,15]. These process parameters jointly govern the extrusion and deposition behavior of the material and, consequently, the printing fidelity and mechanical properties of the printed product [16]. Revealing the effects of different process parameters on printing performance, and optimizing process parameters accordingly, can provide a theoretical basis for the 3D printing of GBR gel products [4,6]. Although these parameters have been individually investigated in other food systems, their interactive effects in starch-rich gel matrices such as GBR have not been systematically investigated, and quantitative optimization remains limited [17,18]. It should be emphasized that 3D printing is expected to improve the texture and palatability of GBR not by altering its intrinsic composition, but by restructuring it into a gel-based architecture with a smooth, uniform surface and a precisely engineered internal porosity, which softens the coarse, gritty mouthfeel associated with intact GBR grains [4].

Existing food 3D printing studies on gel products based on GBR have mostly focused on material formulation design and rheological characterization, with comparatively little attention devoted to process parameters [19,20]. This represents a significant limitation, as process parameters are often more readily adjustable than material formulations in industrial settings and offer a practical pathway for quality control [21]. Therefore, this study systematically investigated the individual and interactive effects of the four aforementioned process parameters on the printing fidelity and hardness of GBR gel. Specifically, single-factor experiments were first conducted to evaluate the effects of individual parameters on printing fidelity and hardness and to define appropriate parameter ranges, followed by response surface methodology (Box–Behnken design) to establish predictive models, elucidate parameter interactions, and optimize process settings.

## 2. Materials and Methods

### 2.1. Materials and Reagents

GBR was prepared following Zhu et al. [2] with minor modifications. Briefly, the brown rice was soaked in distilled water at room temperature (25 ± 2 °C) for 6 h, drained, and germinated at 30 ± 0.5 °C and 85% relative humidity for 32 h, with a water spray time of 12 s applied at 2 h intervals. The prepared GBR samples were freeze-dried to a moisture content of 8.0 ± 0.5% (dry basis), then pulverized in a high-speed grinder and passed through a 100-mesh sieve. The resulting GBR flour was used for preparation of GBR gel. Konjac glucomannan (purity ≥ 95%) and curdlan (purity ≥ 99%) were purchased from Jiahe Food Industry Co., Ltd. (Suzhou, China).

### 2.2. Preparation of GBR Gel and 3D Printing Experiments

**Figure 1:**
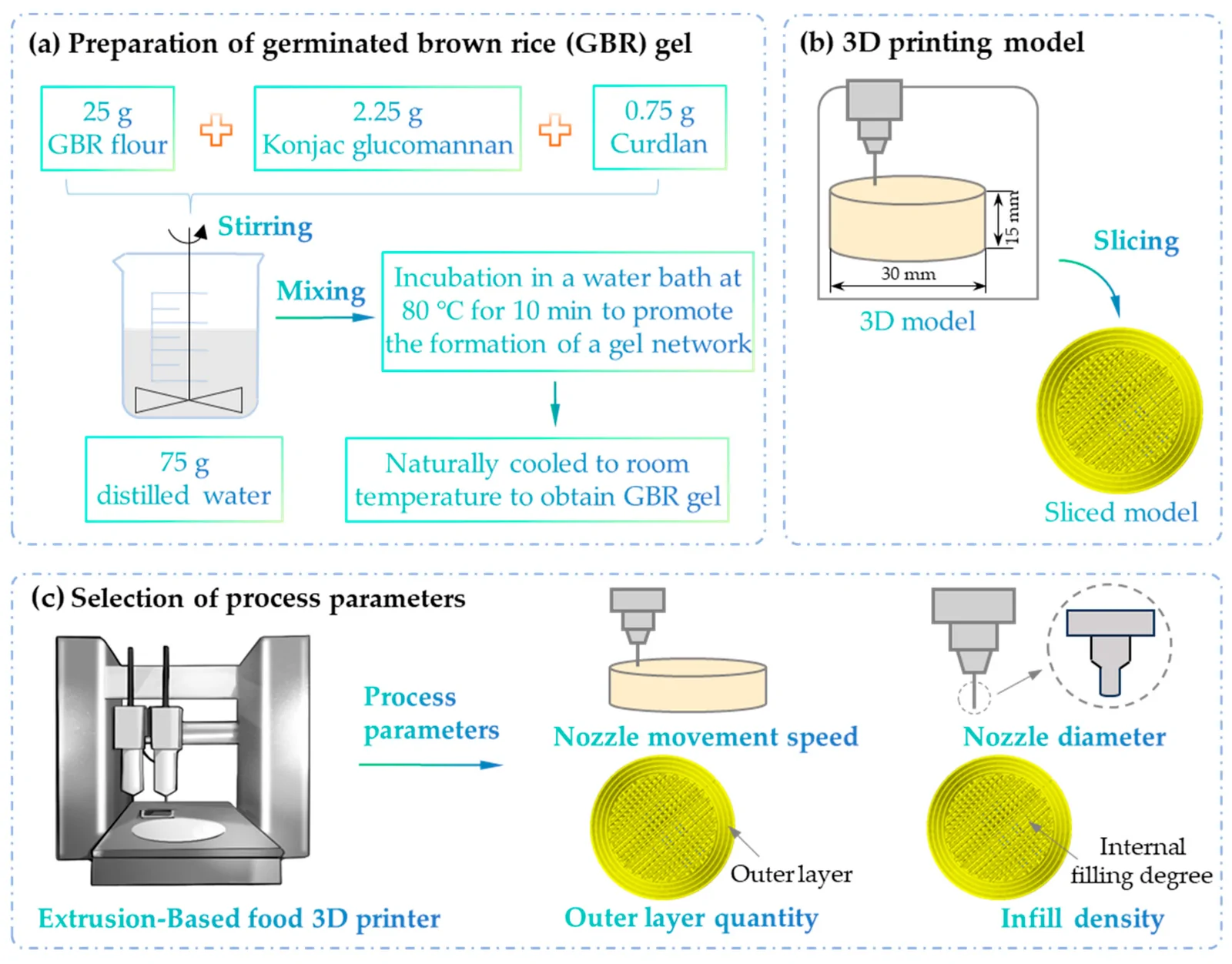
Schematic flowchart of the preparation of GBR gel and 3D printing experiments.

The preparation of GBR gel and the 3D printing experiments are schematically illustrated in Figure 1. Based on preliminary trials regarding gel-forming capacity and system stability, the GBR gel preparation protocol was established as follows: (i) GBR flour (25 g), konjac glucomannan (2.25 g), and curdlan (0.75 g) were dispersed in pre-boiled distilled water (75 g, 98.0 ± 0.5 °C) and homogenized at high speed to obtain a uniform suspension; (ii) the suspension was heated in a water bath at 80 °C for 10 min to induce starch gelatinization and gel network formation; (iii) the sample was subsequently cooled to room temperature naturally to obtain the GBR gel.

The 3D printing experiments of the GBR gel were conducted on an extrusion-based food 3D printer (FOODBOT-D1, Hangzhou Shiyin Technology Co., Ltd., Hangzhou, China). The GBR gel was loaded into the material hopper. A cylindrical test model with a diameter of 30 mm and a height of 15 mm was designed using SolidWorks software (Ver. 2023, Dassault Systèmes, Waltham, MA, USA) and subsequently sliced using UltiMaker Cura (Ver. 4.8, Ultimaker Holding B.V., Utrecht, The Netherlands) prior to printing. The samples were printed using predefined process parameters, including nozzle movement speed, outer layer quantity, infill density, and nozzle diameter.

The following settings were applied in the experiments: the GBR gel was extruded through the nozzle by a piston-driven feeding system at room temperature without additional heating; the layer height and the extrusion (line) width were both set equal to the nozzle diameter; a line infill pattern with the raster orientation alternating at ±45° between adjacent layers was used, with the outer layers printed first to form the peripheral wall; the nozzle-to-platform distance for the first layer was set to approximately 2 mm to prevent the nozzle from scraping the deposited filament while maintaining adequate adhesion; and the printing was performed within 2 h of gel preparation.

### 2.3. Single-Factor Experimental Design

As shown in Table 1, a single-factor experiment was used to investigate the effects of each factor (nozzle movement speed, outer layer quantity, infill density, and nozzle diameter) on the response indicators (printing fidelity and hardness). It should be noted that, due to nozzle availability, a 0.84 mm nozzle was used in place of the originally planned 0.9 mm nozzle to ensure the stability of the GBR gel in 3D printing experiments.

### 2.4. Optimization of Process Parameters via Response Surface Methodology

Nozzle movement speed (*X*_1_), outer layer quantity (*X*_2_), infill density (*X*_3_), and nozzle diameter (*X*_4_) were selected as independent variables, with printing fidelity (*Y*_1_) and hardness (*Y*_2_) of the printed sample as the response variables. Printing fidelity and hardness were chosen as the responses because they represent the two most critical and complementary quality attributes of extrusion-printed foods, namely geometric accuracy and mechanical integrity, and constitute the standard evaluation criteria in comparable process-optimization studies [6,11]. Based on the reasonable ranges of the independent variables determined by the single-factor experiment (*X*_1_: 15–25 mm/s, *X*_2_: 3–5, *X*_3_: 60–100%, *X*_4_: 1.2–1.8 mm), a four-factor three-level Box–Behnken design was adopted, as shown in Table 2. The lower and upper limits of each factor were defined by the onset of printing defects (filament breakage, peripheral bulging, or collapse) in the single-factor tests, ensuring that the design space encompassed the stable printing range. Within this range, the three levels of each factor were evenly spaced around the single-factor optimum, thereby enclosing the combined optimum within the Box–Behnken design space; for infill density in particular, although 80% was optimal in the single-factor tests, the upper level was extended to 100% to bracket the optimum and capture its quadratic behavior, including the risk of overfilling. This design was used to evaluate the individual and interactive effects of the variables on the responses and to determine the optimal process parameters via response surface methodology.

### 2.5. Determination of Response Variables

#### 2.5.1. Printing Fidelity of Printed Samples

Printing fidelity serves as a direct metric for assessing the geometric accuracy of printed samples. Following 3D printing, samples were photographed in a light-controlled chamber to eliminate ambient light interference. The length, width, and height of the central region were then measured using a digital caliper to determine the actual dimensions. Dimensional deviations between the printed sample and the design model were calculated according to Equation (1) [22] for each of the three dimensions. The mean of these three directional fidelity values was then taken as the single printing fidelity value (*Y*_1_), which was used in the statistical analysis.

(1) $$Printing\;fidelity\;\left(\%\right)\;=\;\left(1\;-\;\frac{\left|x_{D}\;-\;x_{0}\right|}{x_{0}}\right)\;\times \;100$$

where $x_{0}$ is the dimension (length, width, or height) of the 3D model, mm; and $x_{D}$ is the corresponding dimension of the printed sample, mm.

#### 2.5.2. Hardness of Printed Samples

Hardness serves as a primary indicator of the mechanical support capacity of printed samples. The hardness measurement was performed according to Zhuang et al. [6]. Briefly, a texture analyzer equipped with a 5 kg load cell (TA.XT. Plus, Stable Micro System Ltd., Surrey, UK) was used. A cylindrical probe (50 mm in diameter) was employed for TPA analysis. The test was carried out at a room temperature of approximately 25 °C. The trigger force was 5 g, and the sample was compressed vertically to 45% of its original height. Pre-test, test, and return speeds were 1, 5, and 5 mm/s, respectively. Hardness was defined as the maximum peak force (g) recorded during the first compression cycle and was extracted from the force-time curve using the instrument software (Texture Exponent, Stable Micro System Ltd., Surrey, UK); measurements were performed at the center of each sample.

### 2.6. Data Processing

All experiments were conducted in triplicate, and the results are expressed as mean ± standard deviation (*n* = 3). Statistical analyses were performed using SPSS 26.0 (IBM Corporation, Chicago, IL, USA). Data were subjected to one-way ANOVA followed by Duncan’s multiple range test to identify differences among treatments. Statistical significance was set at *p* < 0.05. Response surface modeling was performed using a Box–Behnken design in Design-Expert 13.0 software (Stat-Ease, Minneapolis, MN, USA).

## 3. Results and Discussion

### 3.1. Effects of Process Parameters on the 3D Printing Performance of GBR Gel

#### 3.1.1. Effect of Nozzle Movement Speed

**Figure 2:**
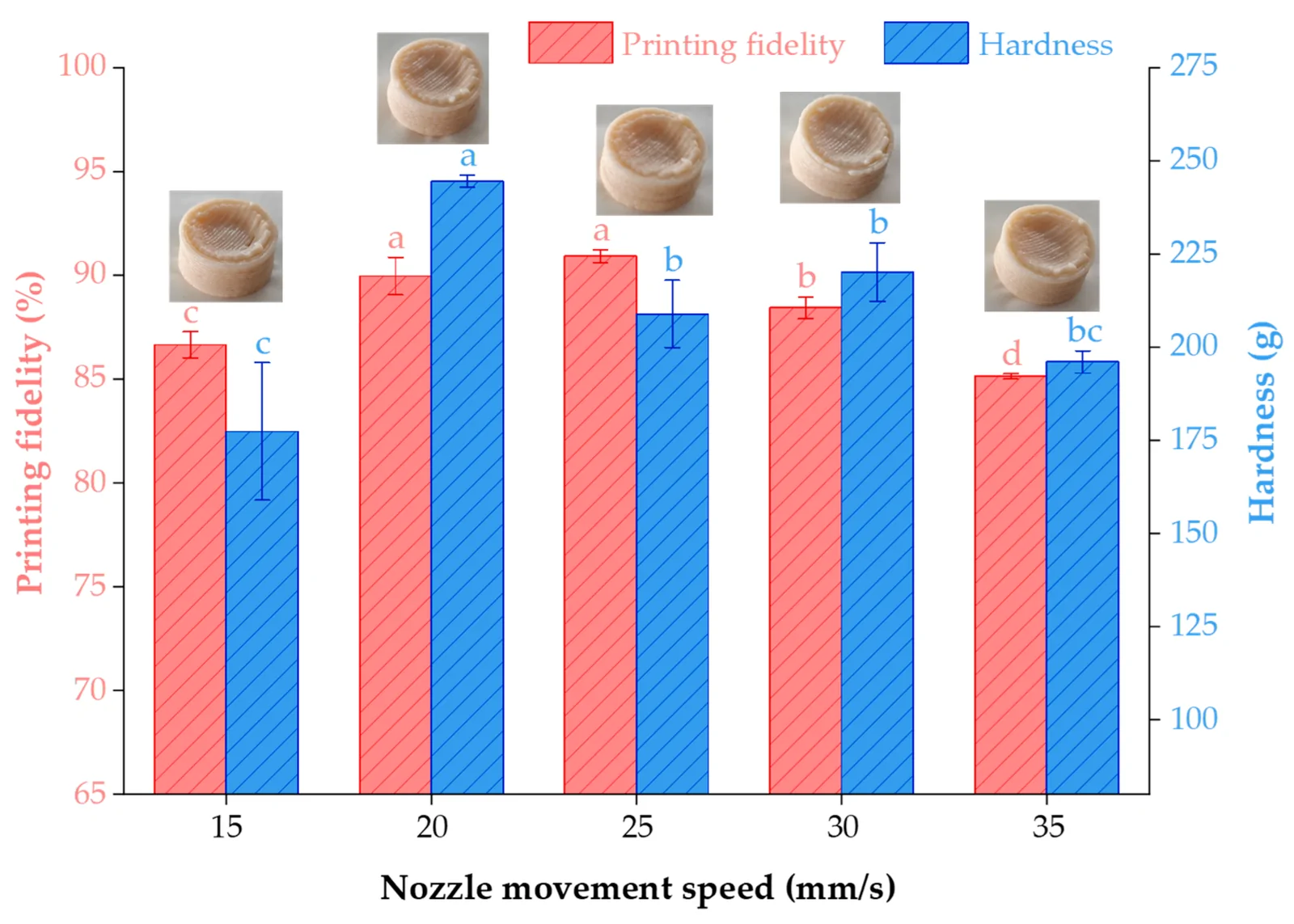
Effect of nozzle movement speed on the printing fidelity and hardness of printed samples. Note: Different lowercase letters above the bars indicate significant differences among treatments (*p* < 0.05). Data are expressed as mean ± standard deviation (*n* = 3).

As shown in Figure 2, nozzle movement speed significantly affected both printing fidelity and hardness (*p* < 0.05). Increasing the nozzle movement speed from 15 to 20 mm/s maximized hardness (244.48 g), while printing fidelity remained high, with only a negligible reduction (*p* > 0.05) compared with that at 25 mm/s. This suggests well-coordinated extrusion output and nozzle movement speed under this condition, facilitating consistent material deposition and enhanced interlayer bonding [6]. Furthermore, when the nozzle movement speed was increased to 25 and 30 mm/s, the hardness of printed samples decreased significantly (*p* < 0.05) compared with the peak at 20 mm/s. This reduction was primarily attributed to insufficient material deposition, which resulted in narrower extrusion lines and diminished interlayer overlap, thereby compromising structural integrity [23]. Upon further increasing the speed to 35 mm/s, printing fidelity reached its minimum value and hardness remained low, indicating that an excessively high nozzle movement speed severely disrupted the continuity of material deposition [24]. Under these conditions, the GBR gel could not achieve stable spreading and sequential stacking, leading to irregular extrusion lines, localized voids, and surface imperfections [25]. Consequently, the printed structure became less compact, with compromised mechanical properties and diminished dimensional accuracy. Overall, nozzle movement speed governs the material deposition rate. Both excessively high and insufficient movement speeds may cause discontinuous filament generation or over-deposition, respectively, which collectively decrease printing accuracy and compromise process stability [26]. Based on a comprehensive assessment of printing fidelity and hardness, a nozzle movement speed of 15–25 mm/s is recommended. This trend is consistent with previous reports for starch-based food systems, in which an intermediate printing speed was likewise found to best coordinate material supply and nozzle traverse [11,24].

#### 3.1.2. Effect of Outer Layer Quantity

**Figure 3:**
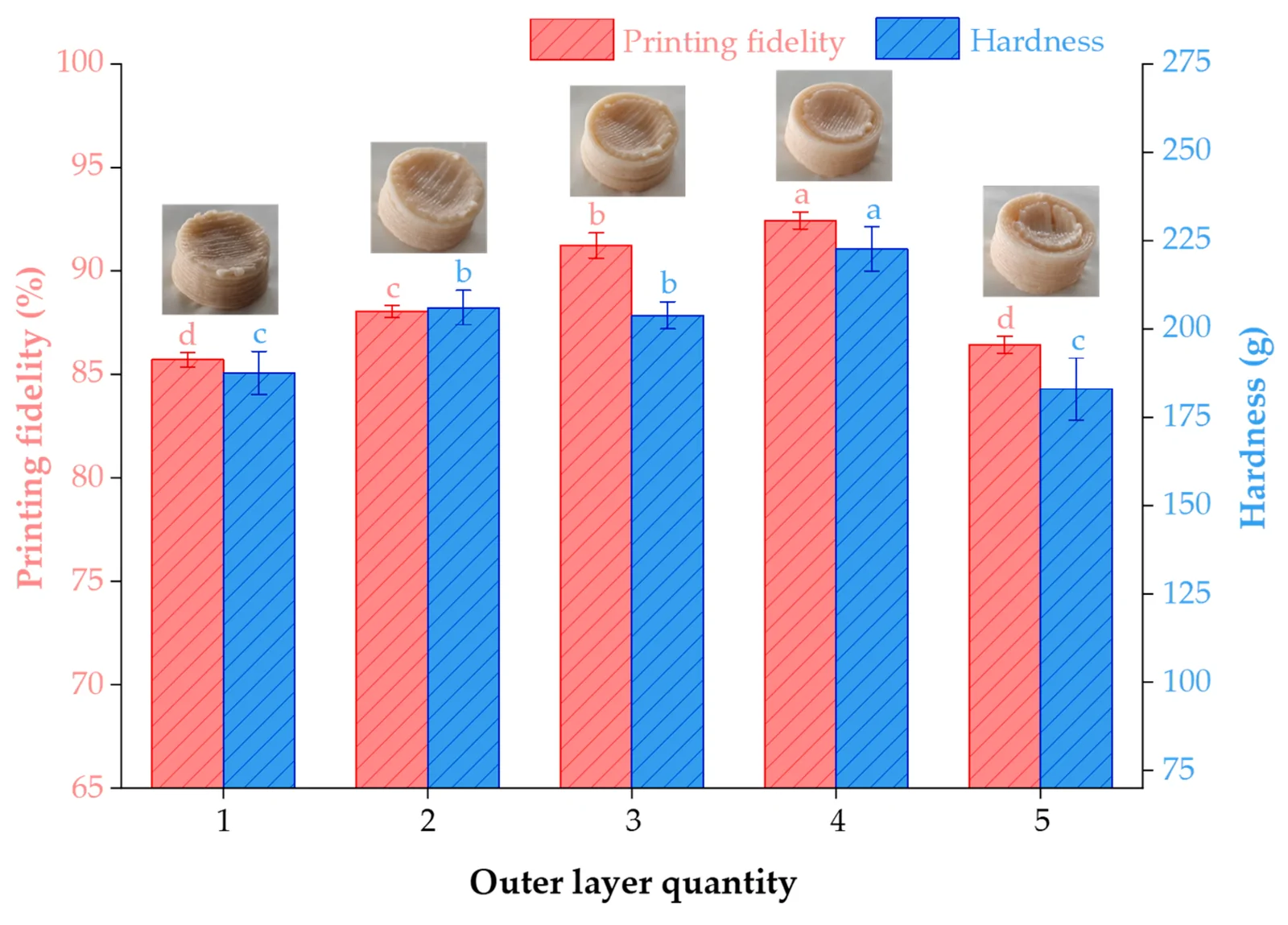
Effect of outer layer quantity on the printing fidelity and hardness of printed samples. Note: Different lowercase letters above the bars indicate significant differences among treatments (*p* < 0.05). Data are expressed as mean ± standard deviation (*n* = 3).

As illustrated in Figure 3, the outer layer quantity significantly affected both printing fidelity and hardness (*p* < 0.05). Increasing the outer layer quantity from 1 to 4 continuously improved both parameters, suggesting that moderately increasing peripheral wall thickness effectively enhanced mechanical support [27]. Within this range, the outer layers formed a complete enclosing framework, constraining deposited beads to conform to the designed path; this geometric confinement effectively restrained sidewall diffusion, peripheral bulging, and local collapse, thereby enhancing overall forming accuracy [25,28]. Simultaneously, improved outer framework stability strengthened interlayer bonding, contributing to enhanced structural strength [25]. However, upon increasing the quantity to 5, both printing fidelity and hardness decreased substantially, indicating that greater peripheral wall thickness does not necessarily improve forming quality [27]. Excessive outer wall layering caused localized material accumulation and incomplete compaction in the peripheral region, producing a less compact outer wall with diminished supporting capacity [25], while also occupying the internal infill space and weakening core support, thereby increasing the risk of localized collapse [28]. This phenomenon underscores that the effect of outer layer quantity exhibits a clear optimum: insufficient wall thickness fails to provide adequate peripheral support, whereas excessive thickness disrupts structural harmony between the outer shell and inner core [29]. Based on comprehensive evaluation of printing fidelity and hardness, an outer layer quantity of 3–5 is recommended.

#### 3.1.3. Effect of Infill Density

**Figure 4:**
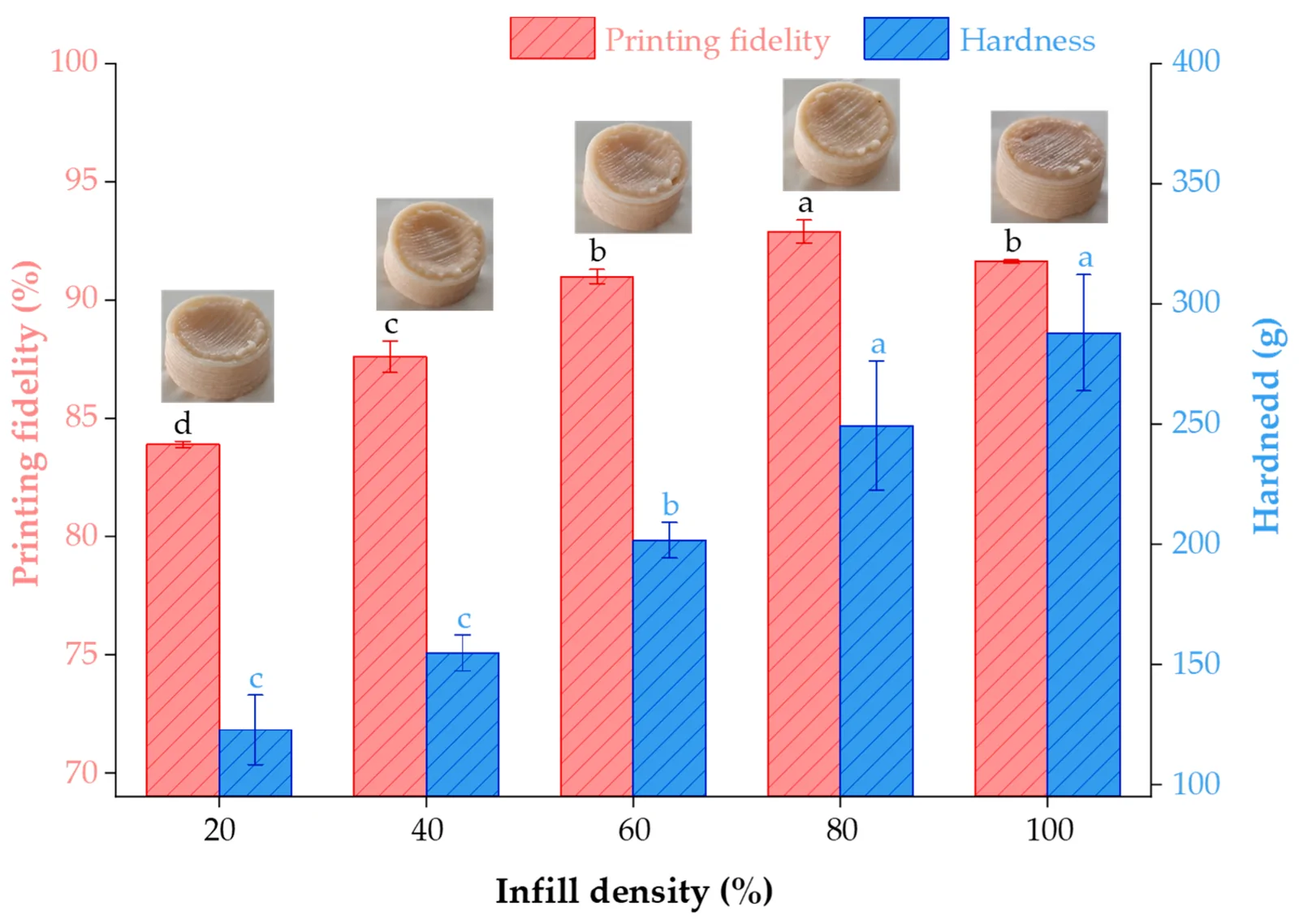
Effect of infill density on the printing fidelity and hardness of printed samples. Note: Different lowercase letters above the bars indicate significant differences among treatments (*p* < 0.05). Data are expressed as mean ± standard deviation (*n* = 3).

As presented in Figure 4, infill density significantly (*p* < 0.05) affected both printing fidelity and hardness. At low infill densities of 20–40%, the internal structure of the samples exhibited considerable porosity with large inter-filament voids, which impeded the formation of a continuous internal support network [30]. Consequently, the core provided inadequate support to the peripheral walls, rendering them susceptible to deformation or localized collapse during deposition and resulting in substantial dimensional deviation and compromised printing fidelity [28,29]. Moreover, limited internal material accumulation yielded low specimen density, with hardness remaining at minimal levels [11].

Upon increasing infill density to 60–80%, internal deposition became more complete and filament continuity improved markedly (*p* < 0.05), establishing a relatively intact three-dimensional support framework. Within this regime, the internal structure effectively resisted deposition-induced pressure on the outer walls, substantially enhancing peripheral stability and enabling the specimens to maintain designed geometry with higher fidelity [11]. Concurrently, hardness increased significantly (*p* < 0.05) as the internal network densified; notably, at 80% infill density, the specimens exhibited superior mechanical properties while preserving high printing fidelity. However, at 100% infill density, although hardness continued to increase due to greater material accumulation, the excessively dense packing constrained internal material flow. This promoted localized stress concentration and peripheral bulging [25], thereby compromising outer wall geometric precision. Based on the observed trends, an infill density of 60–100% is recommended, with 80% representing the optimal compromise between fidelity and hardness. The dominant role of infill density in determining the hardness and geometric precision of printed brown rice-based products is in good agreement with the findings of Huang et al. [31], who identified the infill level as the key variable governing the printing precision and texture of brown rice formulations.

#### 3.1.4. Effect of Nozzle Diameter

**Figure 5:**
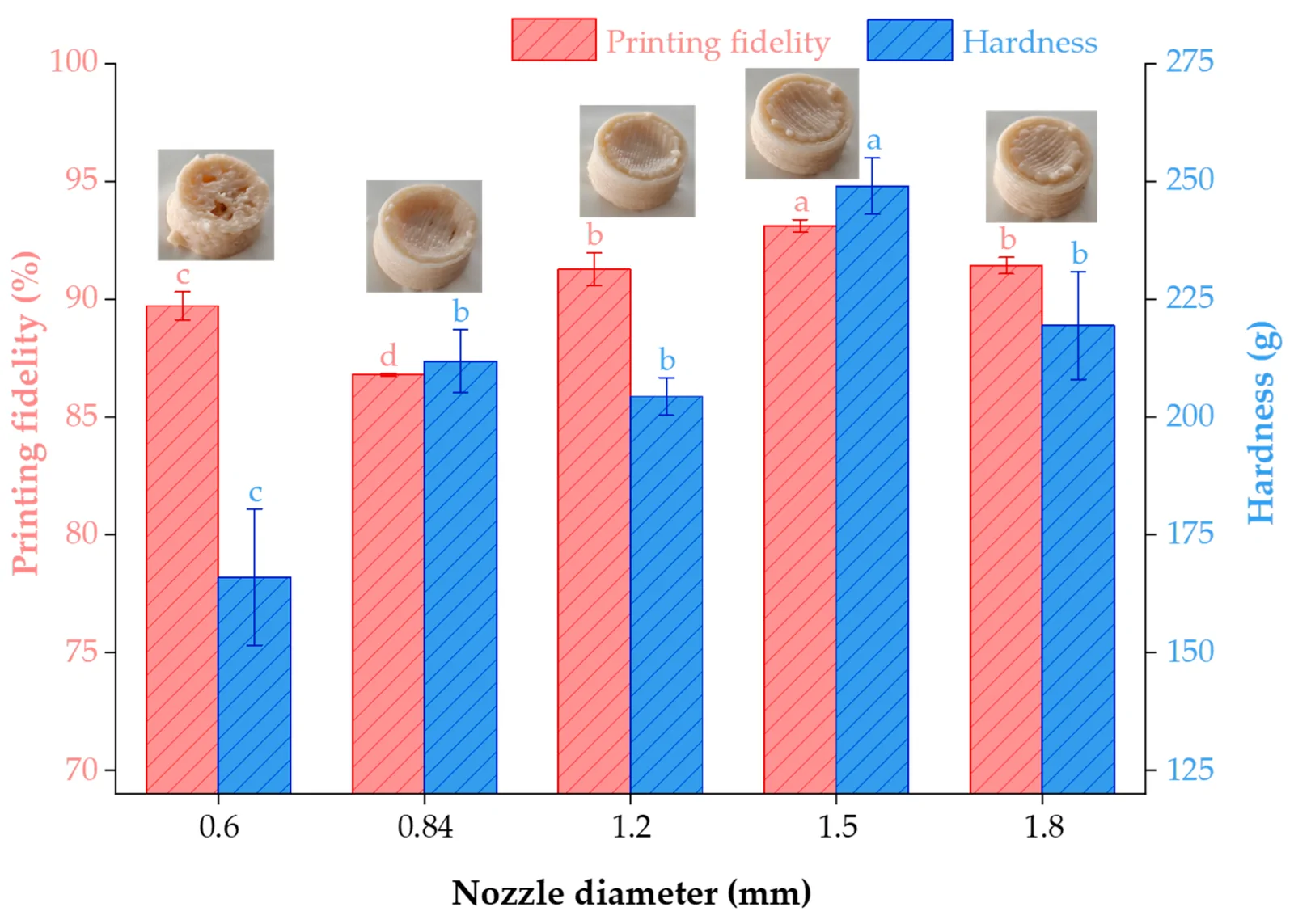
Effect of nozzle diameter on the printing fidelity and hardness of printed samples. Note: Different lowercase letters above the bars indicate significant differences among treatments (*p* < 0.05). Data are expressed as mean ± standard deviation (*n* = 3).

As depicted in Figure 5, nozzle diameter significantly (*p* < 0.05) influenced printing performance. At a nozzle diameter of 0.6 mm, the reduced orifice area induced substantial flow resistance during extrusion, markedly elevating shear stress and causing unstable filament output and intermittent breakage [32]. Under these conditions, the deposited filaments exhibited poor continuity and path integrity, resulting in substantial dimensional deviation. Concurrently, inadequate interlayer overlap diminished structural compactness, and the overall mechanical strength of the specimens remained low [33]. Therefore, a small nozzle diameter is unfavorable for the stable extrusion and forming of medium- to high-viscosity GBR gel systems. Notably, at 0.84 mm, printing fidelity remained suboptimal, suggesting that flow stabilization was not yet fully achieved at this diameter. As the nozzle diameter increased to 1.2–1.5 mm, extrusion resistance decreased, facilitating smoother material flow and uniform filament deposition [34]. Within this range, the deposited filaments maintained geometric stability, interlayer contact area increased, and a structurally intact stacking architecture was established [24]. Both printing fidelity and hardness improved significantly, with 1.5 mm yielding optimal performance. The superiority of the 1.2–1.5 mm range is consistent with the observations of Huang et al. [34] for hydrocolloid-modified brown rice gels, which likewise required nozzles of comparable diameter to achieve stable extrusion and well-defined printed lines.

At 1.8 mm, although extrusion remained smooth, deposition lines became excessively thick, with localized uneven accumulation frequently arising. Consequently, line edges roughened and geometric detail fidelity diminished [24]. Moreover, thick layers were susceptible to non-uniform deformation during cooling, compromising structural uniformity and mechanical integrity [25]. These observations indicate that while larger diameters enhance extrusion efficiency, they are detrimental to fine-feature resolution. Based on comprehensive evaluation, a nozzle diameter of 1.2–1.8 mm is recommended, with 1.5 mm as the optimum for balanced fidelity and hardness.

### 3.2. Interaction Effects of Process Parameters on the Printing Performance of GBR Gel

In Section 3.1, the influence of individual process parameters on the printing performance of GBR gel was systematically investigated. However, in practical 3D printing, the final forming quality is governed by the synergistic interplay of multiple variables. Therefore, drawing upon the level ranges established in the single-factor experiments, response surface methodology was employed to evaluate these interaction effects. The experimental design and results are presented in Table 3.

#### 3.2.1. Interaction Effects of Process Parameters on Printing Fidelity

To elucidate the influence of process parameters on printing fidelity (*Y*_1_, the dependent variable), a multiple regression model was developed with nozzle movement speed (*X*_1_), outer layer quantity (*X*_2_), infill density (*X*_3_), and nozzle diameter (*X*_4_) as the independent variables. The corresponding ANOVA results are presented in Table 4.

As shown in Table 4, the model was highly significant (*p* < 0.01), and the lack-of-fit term was not significant (*p* > 0.05), indicating that the experimental design was reasonable. The coefficient of determination for the reduced quadratic model (R^2^ = 0.8413) and the adjusted coefficient of determination (Adjusted R^2^ = 0.7908) indicate that the model fits the data well, and it could be used to analyze and predict the effects of process parameters on printing fidelity. According to the *F*-value, the order of the impact on printing fidelity was $X_{3}^{2}$, $X_{4}^{2}$, *X*_3_, *X*_2_, *X*_3_*X*_4_, $X_{2}^{2}$, *X*_2_*X*_4_, and the regression model between these parameters and the printing fidelity was obtained using Equation (2)

(2) $$Y_{1}\;=\;92.56\;-\;0.8784X_{2}\;-\;1.04X_{3}\;-\;1.22X_{2}X_{4}\;-\;1.4X_{3}X_{4}\;-\;1.05X_{2}^{2}\;-\;3.54X_{3}^{2}\;-\;1.39X_{4}^{2}$$

where *Y*_1_ is the printing fidelity, %; *X*_2_ is the outer layer quantity; *X*_3_ is the infill density, %; and *X*_4_ is the nozzle diameter, mm.

**Figure 6:**
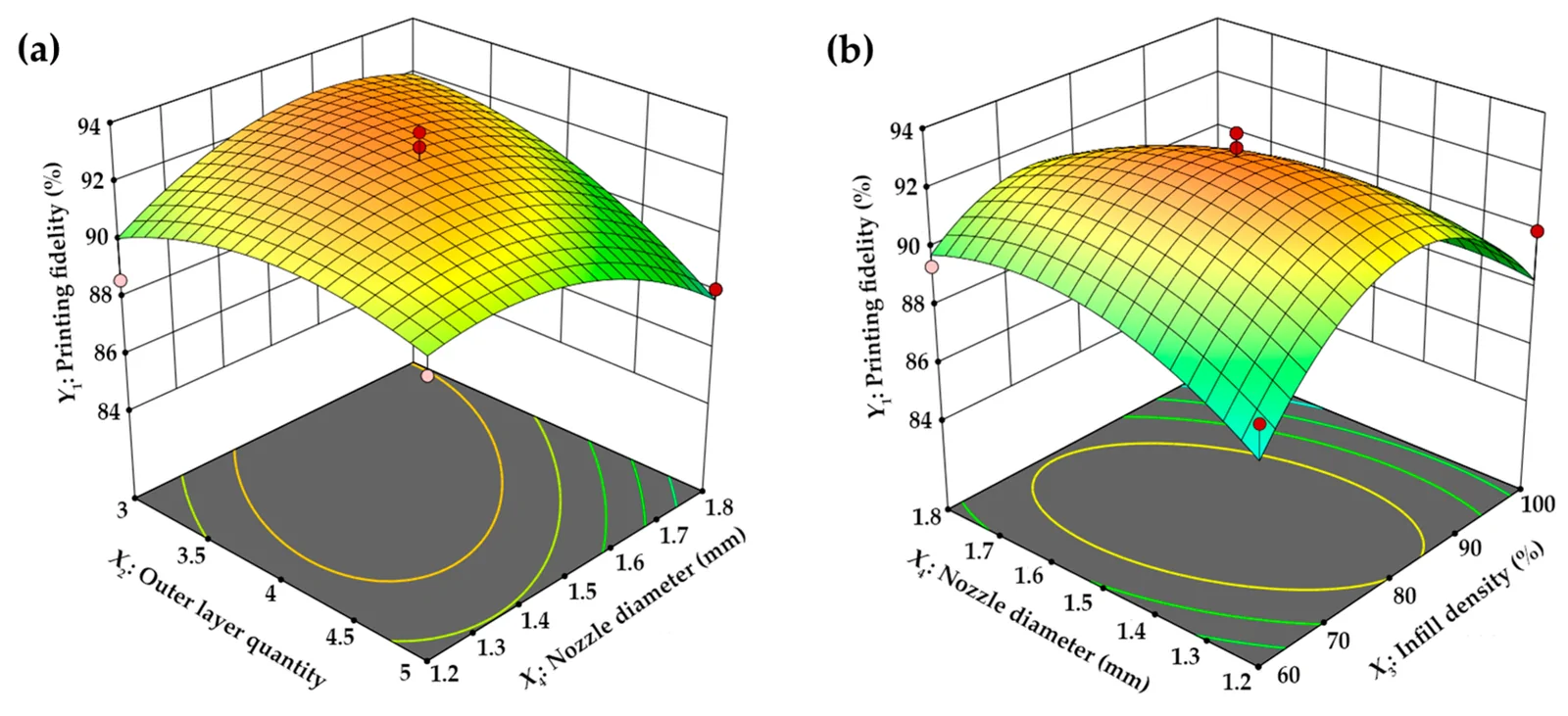
Effects of (**a**) outer layer quantity (*X*_2_) and nozzle diameter (*X*_4_), and (**b**) infill density (*X*_3_) and nozzle diameter (*X*_4_) on printing fidelity.

The response surfaces illustrating the interactive effects of the process parameters on printing fidelity are presented in Figure 6.

As presented in Figure 6a, the interaction between outer layer quantity (*X*_2_) and nozzle diameter (*X*_4_) significantly (*p* < 0.05) affected printing fidelity (*Y*_1_). The response surface exhibited a smooth convex profile, indicating that both factors possess optimal intermediate ranges. Within the synergistic parameter range, stable deposition and uniform inter-bead overlap produced superior fidelity, whereas deviation from the optimum in either direction increased dimensional deviation. Specifically, a small nozzle diameter restricted extrusion flow and produced thin, unstable filaments unfavorable for outer contour formation, while an insufficient outer layer quantity compromised boundary rigidity and exacerbated geometric distortion; moderately increasing the nozzle diameter instead improved material flow, deposition, and inter-bead bonding [35]. In conjunction with an appropriate outer layer quantity, this parameter combination enhanced printing fidelity. However, an excessively large nozzle diameter increased material accumulation and promoted outward filament spreading, rendering the contours susceptible to blurring or expansion [36], while an excessive outer layer quantity similarly amplified this accumulation effect and predisposed the structure to localized deformation. Conversely, combining a large nozzle diameter with an insufficient outer layer quantity left the thick filaments inadequately constrained, also inducing contour deviation. Overall, these parameters interact synergistically by modulating deposition volume, filament geometry, and peripheral support capacity, thereby establishing a well-defined optimal region near their intermediate levels.

As illustrated in Figure 6b, infill density (*X*_3_) and nozzle diameter (*X*_4_) significantly (*p* < 0.05) influenced printing fidelity (*Y*_1_). Building on the mechanisms discussed in Section 3.1.3 and Section 3.1.4, the interaction reflects a coupling between internal support and deposition geometry: an undersized nozzle restricts material deposition and, at low infill densities, prevents the formation of a stable internal network, thereby amplifying the fidelity loss; conversely, a moderate increase in infill density makes material accumulation more continuous, strengthens the internal framework, and allows the outer contours to form more closely along the prescribed path [12]. Similarly, combining a medium nozzle diameter with a medium infill density most effectively establishes the equilibrium between extrusion flow and nozzle traverse, improves inter-bead bonding, and significantly enhances printing fidelity [6]. At the opposite extremes, a large nozzle diameter increases the deposition per unit path and bead width, an effect that is amplified at high infill densities and leads to bead compression, blurred interfaces, and contour deformation [16], whereas pairing a high infill density with a small nozzle generates localized material deficits and voids that are equally detrimental to shape retention.

#### 3.2.2. Interaction Effects of Process Parameters on Hardness

To quantify the effects of process parameters on hardness (*Y*_2_, the dependent variable), a regression model was established with nozzle movement speed (*X*_1_), outer layer quantity (*X*_2_), infill density (*X*_3_), and nozzle diameter (*X*_4_) as the independent variables. The ANOVA results are shown in Table 5.

As shown in Table 5, the regression model was highly significant (*p* < 0.01), while the lack-of-fit term was not statistically significant (*p* > 0.05). Although the lack-of-fit *p*-value (0.0634) is relatively low, suggesting that part of the variation may arise from higher-order effects or gel heterogeneity not captured by the quadratic model, the model is considered adequate for prediction within the design domain. The coefficient of determination of the model (R^2^ = 0.8552) and the adjusted coefficient of determination (Adjusted R^2^ = 0.825) indicate that the model fits the data well, indicating its applicability for analyzing and predicting the effects of process parameters on hardness. According to the *F*-value, the order of the impact on hardness was *X*_4_, $X_{4}^{2}$, *X*_3_, *X*_1_*X*_2_, *X*_1_*X*_3_, and the regression model between these parameters and the hardness was obtained using Equation (3)

(3) $$Y_{2}\;=\;300.19\;+\;31.84X_{3}\;+\;42.61X_{4}\;-\;23.73X_{1}X_{2}\;+\;22.48X_{1}X_{3}\;-\;43.2X_{4}^{2}$$

where *Y*_2_ is the hardness, g; *X*_1_ is the nozzle movement speed, mm/s; *X*_2_ is the outer layer quantity; *X*_3_ is the infill density, %; and *X*_4_ is the nozzle diameter, mm.

**Figure 7:**
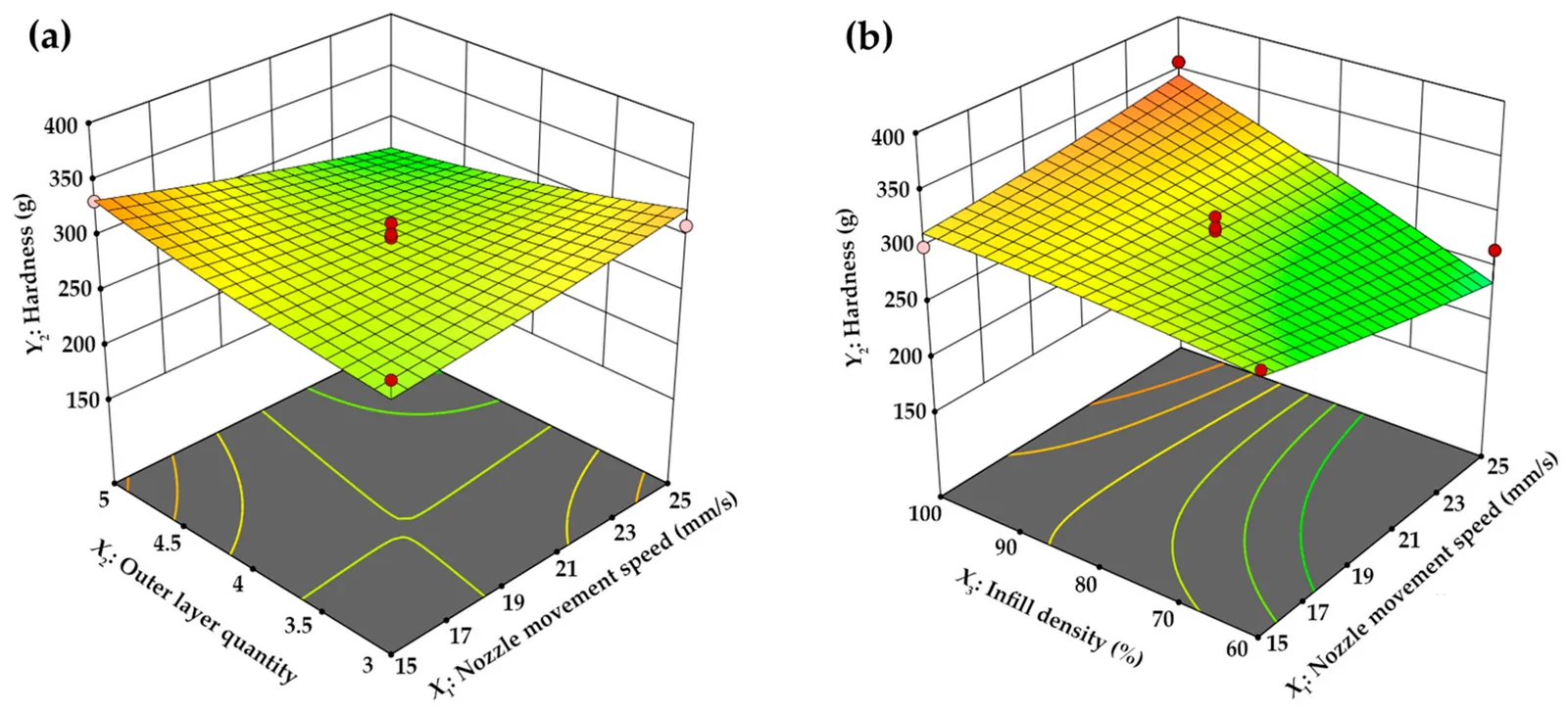
Effects of (**a**) nozzle movement speed (*X*_1_) and outer layer quantity (*X*_2_), and (**b**) nozzle movement speed (*X*_1_) and infill density (*X*_3_) on hardness.

The response surfaces of the process parameters on hardness are shown in Figure 7.

As demonstrated in Figure 7a, nozzle movement speed (*X*_1_) and outer layer quantity (*X*_2_) exhibited a significant interactive influence on hardness (*Y*_2_). At a low nozzle movement speed of 15 mm/s, the hardness increased with the quantity of the outer layer due to the resulting boundary support, which further enhanced the load-bearing capacity of the structure [37]. However, as the nozzle movement speed increased, the relative material extrusion decreased, localized gaps formed, reducing structural compactness and gradually lowering hardness. At a low outer layer quantity (3 layers), the hardness increased slightly with nozzle movement speed over the same interval, as the reduced deposition per unit path alleviated localized accumulation and peripheral bulging of the thin wall and produced a more regular, better-compacted periphery [25]. Conversely, at a high outer layer quantity (5 layers), hardness decreased markedly as the nozzle movement speed increased from 15 to 25 mm/s; this was because the greater material demand of the thick peripheral wall per unit path could not be met at elevated speeds, resulting in diminished inter-bead overlaps and the formation of localized voids, which compromised structural compactness [38]. Overall, a thicker peripheral wall benefits hardness only when the material supply per unit path is sufficient, i.e., at low-to-moderate nozzle movement speeds, indicating that the two parameters must be tuned jointly.

As illustrated in Figure 7b, nozzle movement speed (*X*_1_) and infill density (*X*_3_) also exhibited a significant interactive effect on hardness (*Y*_2_). The response surface ascended continuously with increasing infill density, whereas the effect of nozzle movement speed was comparatively weak and was modulated by the levels of the other factors, indicating that hardness was governed primarily by the combined influence of material deposition volume and interlayer bonding integrity. At high infill densities, the fully developed load-bearing scaffold and the expanded interlayer contact area formed a denser microscale network [31], and the abundant material supply and close bead contact allowed a moderately increased movement speed to stretch the bead and increase the inter-bead contact pressure, with hardness therefore increasing slightly with nozzle movement speed and the printed constructs consistently exhibiting superior hardness [37]. At low infill densities, by contrast, the increased void fraction compromised the structural framework, rendering hardness largely dependent on localized inter-bead adhesion [39]; an elevated nozzle movement speed further reduced the deposition per unit path and the inter-bead overlap, producing a more pronounced hardness decline, whereas a reduced nozzle movement speed allowed the relatively abundant material supply to improve interlayer contact and partially mitigate the hardness loss [30]. Overall, increasing the infill density promoted structural compactness, whereas the effect of nozzle movement speed was governed by its interplay with material supply, which is consistent with the findings of Barrios-Rodríguez et al. [40] regarding 3D printing with rice protein.

### 3.3. Optimization and Verification of Process Parameters

To identify the optimal process parameters for the extrusion-based 3D printing of GBR gel, Design-Expert 13.0 software was employed to optimize the experimental variables. In the numerical optimization module, the optimization goals were defined as follows: printing fidelity (*Y*_1_) was set to “maximize”, hardness (*Y*_2_) was constrained “in range” (i.e., within the values experimentally observed), and all four independent variables were restricted to the experimental design range. Among the candidate solutions generated by the software, the solution achieving the highest desirability (i.e., the greatest predicted printing fidelity) was selected as the optimum. Accordingly, the “in range” constraint for hardness corresponded to the experimentally observed range of 161.63–357.51 g (Table 3), within which any value was considered acceptable; hardness was not maximized, because an excessively hard construct would be inconsistent with the goal of improving the texture and palatability of GBR products. The resulting optimal parameter combination is summarized in Table 6.

To validate the reliability of the optimized process parameters, confirmatory experiments were performed. Accounting for equipment resolution and practical constraints, the theoretically predicted optimum was rounded to the nearest experimentally achievable settings: a nozzle movement speed of 25 mm/s, an outer layer quantity of 4, an infill density of 76%, and a nozzle diameter of 1.5 mm. Triplicate validation runs were conducted under these conditions, yielding a printing fidelity of 94.64 ± 1.14% and a hardness of 314.16 ± 25.70 g. The coefficients of variation in the two responses were approximately 1.2% and 8.2%, respectively. The larger variability of hardness is mainly attributable to the inherent heterogeneity of the gel network and to layer-to-layer bonding fluctuations, which are typical of extrusion-printed starch-rich gels [25]. Relative to the model predictions at the optimum, the deviations were 1.80% and 0.50%, respectively, demonstrating satisfactory agreement between prediction and validation. These results indicate that the models possess good predictive performance within the investigated experimental domain; however, as with any response-surface model, extrapolation beyond the tested ranges requires further verification. From a practical standpoint, the optimized parameter set can be directly adopted for the present konjac-glucomannan/curdlan-reinforced GBR gel on desktop extrusion-based 3D printers used in the current study, and it provides a reference parameter range for similar starch-based food gels; transfer to formulations with substantially different rheological properties or to other printer types should nevertheless be accompanied by confirmatory printing tests.

## 4. Conclusions

This study systematically elucidated the individual and interactive effects of process parameters (nozzle diameter, nozzle movement speed, infill density, and outer layer quantity) on the printing fidelity and hardness of GBR gel. Whereas printing fidelity was governed mainly by the internal architecture, with infill density as the dominant factor and outer layer quantity and nozzle diameter contributing through significant linear, quadratic and interaction effects, hardness was determined primarily by extrusion geometry and infill density. The significant two-way interactions further demonstrate that these parameters must be tuned together rather than in isolation. The optimized parameters (nozzle movement speed of 25 mm/s, outer layer quantity of 4, infill density of 76%, and nozzle diameter of 1.5 mm) were experimentally validated with prediction errors below 2%, confirming the reliability of the models within the investigated domain. It should be emphasized that these findings are specific to the present GBR gel formulation and printer configuration, and their extension to other starch-rich systems requires further validation. Future work should therefore combine the optimized parameters with systematic sensory and nutritional evaluation, elucidate the effects of post-processing treatments on the structure and texture of the printed products, and extend the methodological framework to other germinated cereals and starch-rich gel systems, so as to facilitate the broader industrial application of 3D printing in functional food development.

## Figures and Tables

**Table 1:** Single-factor experimental design scheme.

Experimental Factors	Levels				
Nozzle movement speed (mm/s)	15	20	25 ^#^	30	35
Outer layer quantity	1	2	3 ^#^	4	5
Infill density (%)	20	40	60 ^#^	80	100
Nozzle diameter (mm)	0.6	0.84	1.2 ^#^	1.5	1.8

Note: Values marked with “#” indicate fixed values for the other three factors when one factor is used as a test variable. For example, when exploring the impact of nozzle movement speed on the response indicators, the values of the other three factors (outer layer quantity, infill density, and nozzle diameter) are fixed at 3, 60, and 1.2, respectively.

**Table 2:** Factors and levels of response surface experiments.

Experimental Factors	Levels		
	−1	0	1
Nozzle movement speed (mm/s)	15	20	25
Outer layer quantity	3	4	5
Infill density (%)	60	80	100
Nozzle diameter (mm)	1.2	1.5	1.8

**Table 3:** Experimental scheme and test results.

Test No.	Independent Variables				Response Variables	
	Nozzle Movement Speed *X*_1_ (mm/s)	Outer Layer Quantity *X*_2_	Infill Density *X*_3_ (%)	Nozzle Diameter *X_4_* (mm)	Printing Fidelity *Y*_1_ (%)	Hardness *Y*_2_ (g)
1	−1	−1	0	0	93.4347	307.016
2	−1	0	1	0	86.4667	301.239
3	0	−1	−1	0	89.4754	241.634
4	−1	0	0	−1	92.2493	233.305
5	1	0	−1	0	89.6208	266.835
6	0	0	1	−1	90.0333	247.657
7	0	0	−1	1	89.4072	270.541
8	0	−1	0	−1	88.6620	235.812
9	1	0	0	1	91.7347	298.933
10	0	0	0	0	91.3615	303.294
11	1	−1	0	0	93.7616	310.789
12	−1	1	0	0	90.4833	332.374
13	1	0	1	0	88.4333	357.508
14	1	1	0	0	90.5957	241.209
15	1	0	0	−1	89.7379	206.570
16	0	−1	0	1	91.4782	302.303
17	0	−1	1	0	87.6833	350.500
18	0	0	0	0	92.4969	296.425
19	0	0	0	0	93.1055	298.194
20	0	1	0	−1	90.1830	201.308
21	0	0	0	0	93.5853	281.490
22	0	1	−1	0	89.1765	267.339
23	−1	0	0	1	90.2241	288.230
24	−1	0	−1	0	89.0275	300.500
25	0	1	1	0	85.3833	333.953
26	0	0	0	0	92.2448	300.075
27	0	0	0	0	93.0910	313.107
28	0	0	1	1	85.0500	299.650
29	0	0	−1	−1	88.7707	161.631
30	0	1	0	1	88.1333	337.938

**Table 4:** ANOVA results for the reduced quadratic model for printing fidelity.

Source	Sum of Squares	Degrees of Freedom	Mean Square	*F*-Value	*p*-Value
Model	131.71	7	18.82	16.66	<0.0001 **
*X*_2_	9.26	1	9.26	8.20	0.0090 *
*X*_3_	12.87	1	12.87	11.39	0.0027 **
*X*_2_ *X*_4_	5.92	1	5.92	5.24	0.0320 *
*X*_3_ *X*_4_	7.90	1	7.90	6.99	0.0148 *
*X*_2_^2^	7.66	1	7.66	6.78	0.0162 *
*X*_3_^2^	87.53	1	87.53	77.48	<0.0001 **
*X*_4_^2^	13.61	1	13.61	12.04	0.0022 **
Residual	24.85	22	1.13		
Lack-of-fit	21.73	17	1.28	2.05	0.22
Pure error	3.12	5	0.6249		
Total deviation	156.56	29			
Correlation coefficient	R^2^ = 0.8413, Adjusted R^2^ = 0.7908				

Note: ** *p* < 0.01, * *p* < 0.05. For the reduced quadratic model, the insignificant terms (*p* > 0.05) were not included in the table.

**Table 5:** ANOVA results for the reduced quadratic model for hardness.

Source	Sum of Squares	Degrees of Freedom	Mean Square	*F*-Value	*p*-Value
Model	51,663.19	5	10,332.64	28.34	<0.0001 **
*X*_3_	12,162.05	1	12,162.05	33.36	<0.0001 **
*X*_4_	21,786.66	1	21,786.66	59.76	<0.0001 **
*X*_1_ *X*_2_	2253.31	1	2253.31	6.18	0.0203 *
*X*_1_ *X*_3_	2022.03	1	2022.03	5.55	0.0270 *
*X*_4_^2^	13,439.14	1	13,439.14	36.87	<0.0001 **
Residual	8749.03	24	364.54		
Lack-of-fit	8216.88	19	432.47	4.06	0.0634
Pure error	532.15	5	106.43		
Total deviation	60,412.22	29			
Correlation coefficient	R^2^ = 0.8552, Adjusted R^2^ = 0.825				

Note: ** *p* < 0.01, * *p* < 0.05. For the reduced quadratic model, the insignificant terms (*p* > 0.05) were not included in the table.

**Table 6:** Optimization and verification results of process parameters.

	Process Parameters	Goal	Predicted Value
Independent Variables	Nozzle movement speed *X*_1_ (mm/s)	In range	24.766
	Outer layer quantity *X*_2_	In range	3.444
	Infill density *X*_3_ (%)	In range	76.148
	Nozzle diameter *X_4_* (mm)	In range	1.615
Response Variables	Printing fidelity *Y*_1_ (%)	Max	92.95
	Hardness *Y*_2_ (g)	In range	312.5

## Data Availability

The original contributions presented in the study are included in the article; further inquiries can be directed to the corresponding authors.
